# Effect of different implant positions for two implant-retained mandibular overdenture: a retrospective 5-years radiographic evaluation of the circumferential peri-implant bone loss and posterior ridge resorptive changes

**DOI:** 10.1186/s12903-024-04871-w

**Published:** 2024-09-30

**Authors:** Khloud Ezzat Mourad, Radwa Mohsen Kamal Emera, Ahmed Habib

**Affiliations:** Department of Prosthodontics, Faculty of Dentistry, Mansoura University, Mansoura, Dakahlia, Egypt

**Keywords:** Implant overdenture, Peri-implant bone loss, Posterior ridge resorption

## Abstract

**Background:**

Studies did not recommend which position for implant overdenture poses the lowest biomechanical risk and the least chance of peri-implant bone loss and ridge resorption for those who might need a mandibular two-implant overdenture. The study objectives were to investigate the impact of implant position, in lateral incisors or canine positions, on peri-implant bone loss and posterior ridge resorption.

**Methods:**

Fifty patients with mandibular two-implants were recalled and divided according to the implant position into two groups (group L: implants in lateral incisor positions and group C: implants in canine positions). The circumferential peri-implant bone level and posterior ridge resorption were assessed at implant insertion (T0), one year later (T1), and five years later (T5) using the follow-up CBCT. Data were analyzed using the Statistical Package of Social Science (SPSS) program. A Mann-Whitney test was used to compare two different groups. Paired groups were compared using the Wilcoxon signed-rank test. The threshold of significance is fixed at a 5% level (p-value).

**Results:**

Significant differences in the vertical bone loss between groups appeared at (T5 - T1) (Mann Whitney test, (*P* = 0.01)) and at (T5 - T0) (Mann Whitney test, (*P* = 0.005)), and a significant difference in horizontal bone loss between groups was found at (T1 - T0) (Mann Whitney test, (*P* = 0.041)) and (T5 - T1) (Mann Whitney test, (*P* = 0.041)). Also, there were significant differences over the evaluation period between groups at certain points along the ridge at M1 (Mann Whitney test, (*P* = 0.021)), M3 (Mann Whitney test, (*P* = 0.008)), and M4 (Mann Whitney test, (*P* = 0.015)).

**Conclusions:**

According to the findings of this clinical study, the placement of implants in the lateral incisor position for two implant-retained overdentures is a viable choice. In comparison to the canine position, the lateral incisor position demonstrated superior peri-implant responses, which could potentially enhance the longevity of the implants. Furthermore, the placement of implants in the lateral incisor position can promote a more even distribution of stress and help mitigate posterior ridge resorption. Conversely, implants in the canine position may cause a seesaw effect and result in greater posterior ridge resorption.

**Clinical Trial Registry Number:**

(NCT06055842) (13/03/2024).

## Background

Dentists have long faced a challenging task in rehabilitating a completely edentulous mandible. Patients who suffer from moderate to severe mandibular alveolar bone resorption often face functional and psychological difficulties with traditional dentures due to a lack of stability and retention. Fortunately, implant-retained overdentures have become the standard for treating a completely edentulous mandible. With high predictability and longevity, dental implants boast an impressive success and survival rate of over 95.5% [1]. This approach offers patients a higher level of satisfaction, comfort, and overall quality of life when compared to traditional full dentures [2, 3].

The debate surrounding the necessary number of implants for mandibular overdentures seems to have been settled. The McGill and York international consensus states that two inter-foraminal site implants are sufficient for achieving optimal stability and chewing function [4, 5]. This approach has gained support from recent dental research, which has found that the 2-implant overdenture is an effective treatment for edentulous mandibles [6–8]. Studies show that the inter-foraminal region, particularly at the canine level, is the ideal spot for the two implants. However, alternative implant placements may be considered if bone availability is restricted. In such cases, a dentist may opt to place the implants at the mandibular symphysis (at the lateral incisor level) [9–11].

Different attachment types can be used for such overdentures, including bars of different designs, balls, and magnetic and resilient telescopic attachments [12]. Compared to splinted attachments, non-splinted attachments offer several advantages. They are cost-effective, easier to clean, and have fewer complications. In addition, they are indicated for distal implant location and can be used in patients with a pointed arch who do not have sufficient tongue space for bars [13, 14].

When attaching a denture to an implant, there is a chance of movement between them, especially when a resilient attachment is utilized. However, most of the movement is absorbed by the alveolar ridge, which can lead to decreased mucosal resilience [15, 16]. When force is applied to an implant-retained overdenture, the implants act as a fulcrum, causing subsequent rotational movement around the implant, with both anterior and posterior lever arms. If the force is applied to the posterior lever arm, the primary and secondary bearing areas of the overdenture will resist it. However, if the force is applied to the anterior lever arm, such as incisive movements, it may result in more noticeable rotation. To reduce tipping stresses on the overdenture, it is recommended to shift the implants from the canine to the lateral incisor position. This reduces the effective anterior lever arm, resulting in less noticeable rotation [17].

According to in-vitro studies, anterior implant positions for two-implant overdentures led to higher stress concentration in the posterior mandible regions [9, 18, 19]. However, the studies did not recommend which position, in the lateral incisors or canines, poses the lowest biomechanical risk and the least chance of peri-implant bone loss and ridge resorption for those who might need a mandibular two-implant overdenture. This implies that there might be varying rates of bone loss in different implant positions, which could have significant implications for research and clinical practice.

Peri-implant marginal bone loss is one of the most traditionally used criteria to evaluate the success of osseointegrated dental implants and can predict stress or overloading on implants [20]. Cone beam computed tomography (CBCT) is considered a useful method for evaluating bone changes because it presents dimensions that are compatible with the actual size and does not cause distortion or overlapping [21]. CBCT is also used to precisely identify and assess bone changes in edentulous sites [22]. Following the insertion of a denture, modifications can impact the hard and soft tissues of the residual ridge. This is due to the compression of the soft tissue mucosa beneath the denture base, which can have an impact on the blood supply responsible for delivering nutrients and removing waste products from the bone. As a result, resorption may occur [23]. With this in mind, the purpose of this investigation was to evaluate the effects of implant placement in the lateral incisor and canine regions on bone changes following the insertion of an implant-retained overdenture. The primary outcome of the research was to observe changes in the level of peri-implant bone, while the secondary outcome was the resorption of the posterior ridge in the mandible. The null hypothesis of the study posits that there are no differences between the two implant positions concerning peri-implant bone loss or resorptive changes in the posterior mandibular ridge.

## Methods

**Fig. 1:**
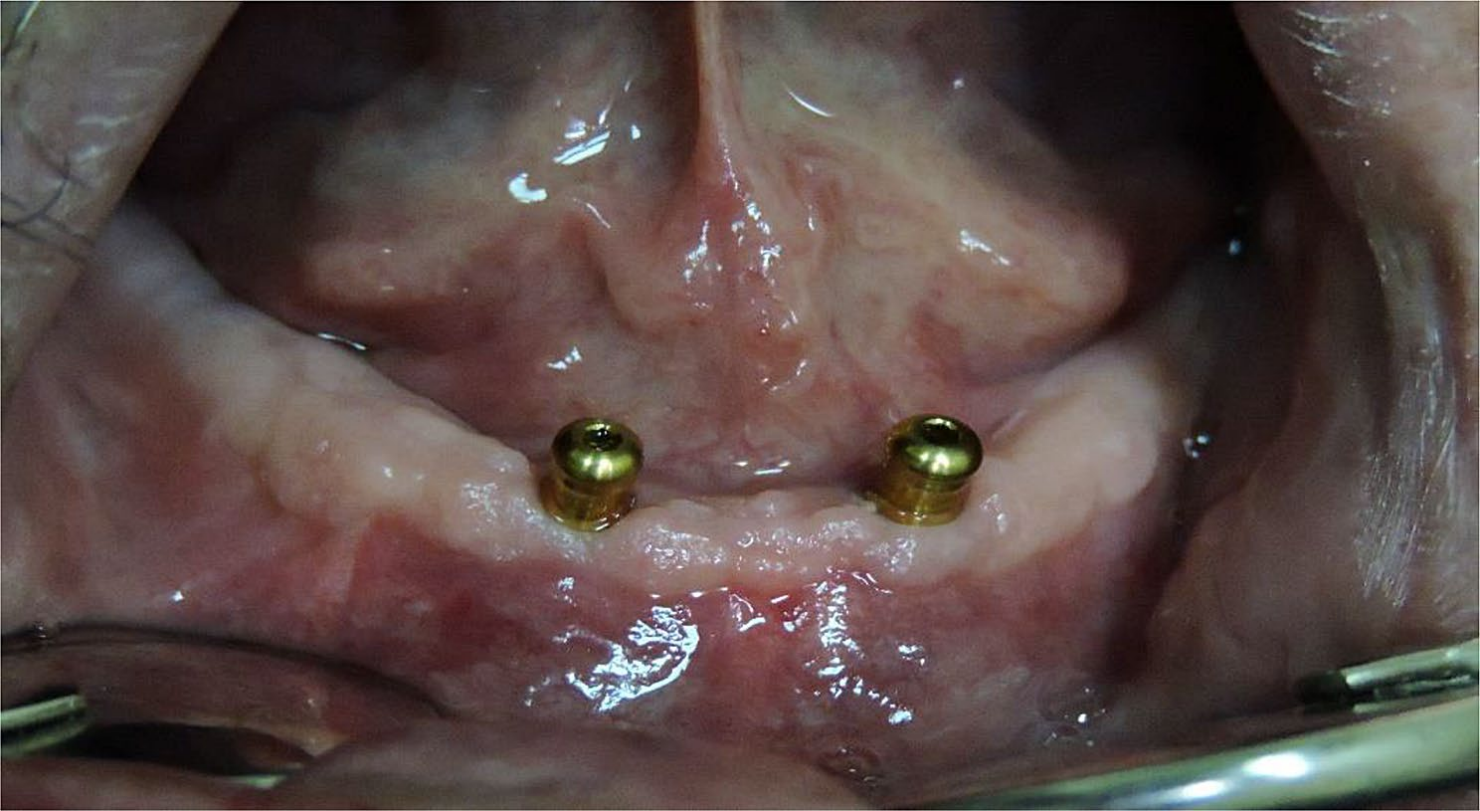
(Group L) Implants in the lateral incisors’ positions

**Fig. 2:**
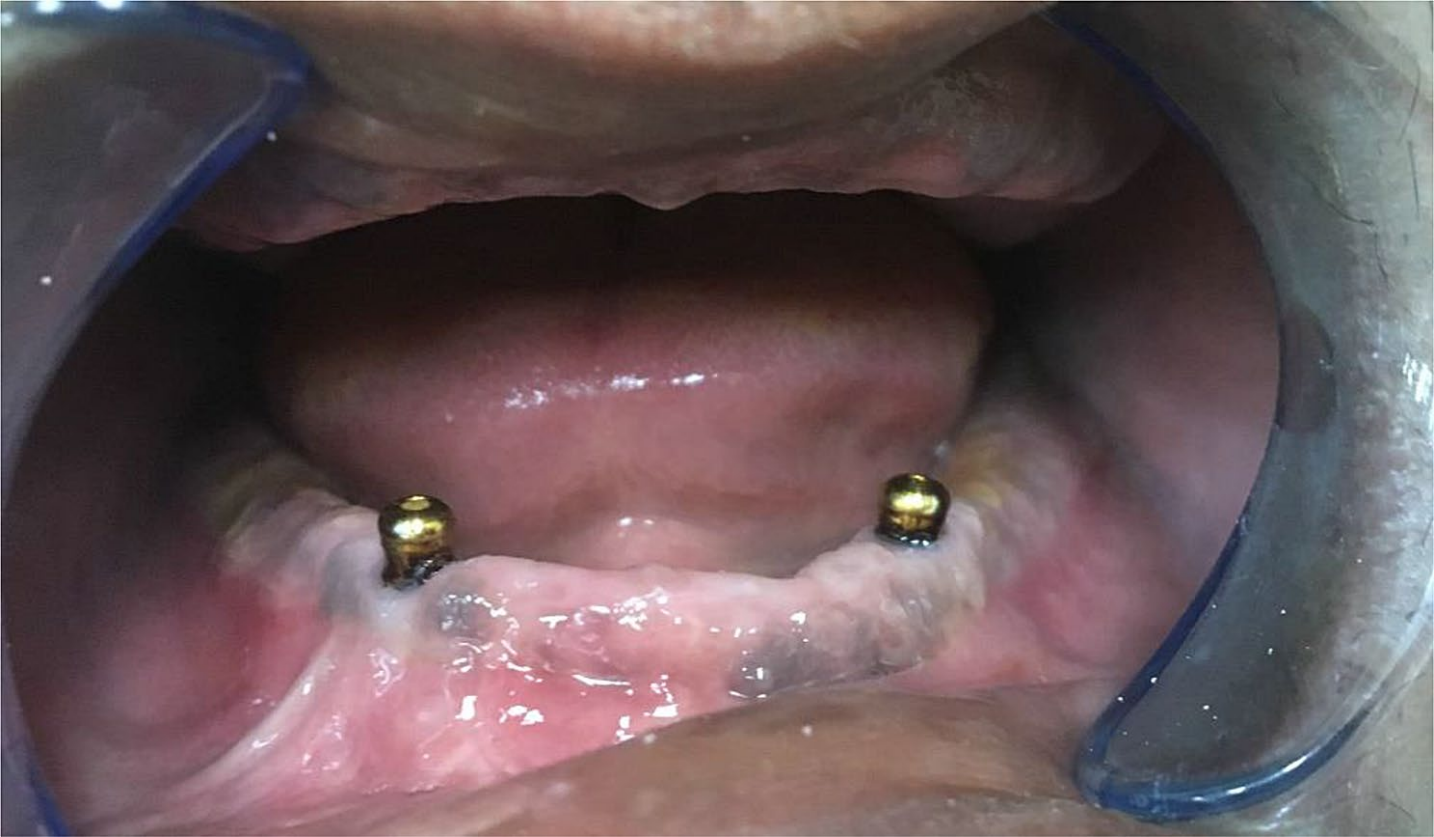
(Group C) Implants in the canines’ positions

Patients for this retrospective study were selected from clinical records in the prosthodontic department. The patients were chosen based on the following criteria: (a) implant insertion before August 2018; (b) availability of CBCT follow-up radiographs at the time of insertion (baseline), after one year, and five years; (c) regular check-ups for prosthetic maintenance (occlusal adjustments, need for relining, and replacement of the attachment matrix) of the prosthesis. All included patients were well-maintained and were recalled every six months for the first two years and then yearly. Individual records were excluded if: [1] only panoramic radiographs were available; [2] there were no follow-up radiographs; or [3] the patient did not attend the follow-up visits. The study included 50 male patients who had received a mandibular overdenture on two implants and a maxillary complete denture at the department clinic since 2017. The 50 patients were divided into two groups, each comprising 25 patients. Group L comprised patients with two implants in the lateral incisors area (Fig. 1), while group C comprised patients with two implants in the canine area (Fig. 2).

From the selected patients’ records, all of the selected patients in this study were completely edentulous and met the following criteria: they had healthy mucosa covering their maxillary and mandibular residual alveolar ridges, without any remaining roots or local inflammation. Additionally, they had sufficient residual alveolar bone quantity (height and width) and quality (normal trabecular pattern) in the inter-foraminal area to receive implants. These criteria were verified for each patient using 3-D panoramic radiographs. The patients also had Angel’s skeletal Class I maxillomandibular relation [24] and sufficient restorative space detected by tentative jaw relation. They were healthy and free from any systemic diseases that may affect bone health, such as uncontrolled diabetes mellitus and osteoporosis. Patients with a history of parafunctional habits (bruxism and clenching), smoking and alcoholism, local or general contraindications for surgical procedures, TMJ or neuromuscular disorders, and a history of radiation therapy in the head and neck region were excluded from the study.

The sample size for this study was calculated on the G-power program (version 3.1.9.7). According to the results of a previous study [22], a sample size of 48 participants and 2 extra cases anticipating the dropout (25 in each group) was selected to yield 95% power with an effect size of 1.065. The study was approved by the ethical committee of the Faculty of Dentistry with approval number (M0108023RP). The surgical and prosthetic steps were as follows: all patients had received conventional maxillary and mandibular complete dentures. The mandibular complete denture for each patient has been duplicated into clear acrylic resin to construct a conventional surgical stent. According to the patient’s group of implant locations, each patient had received two mandibular implant fixtures (Dentium SuperLine, with implant characteristics S.L.A. surface treatment, double-threaded, tapered body design, full-length cutting flutes, Dentium, Co. Ltd., Korea) of 12 mm length and 3.4 mm diameter using the two stages of the surgical protocol. After 3 months of the osseointegration period, the positioner abutments (Dentium, Co. Ltd., Korea) (3.5 mm diameter and 3 mm height) have been screwed into position. The positioner socket set was then incorporated into the denture using direct pickup procedures. The occlusion was adjusted to a bilateral balanced occlusion [9, 25]. Instructions for performing proper hygiene were provided, and the recall visits were scheduled.

### Evaluation of the circumferential peri-implant bone level changes

Using a CBCT and the approach outlined by Elsyad et al., the changes in the peri-implant bone level on each implant face were evaluated [26]. To achieve a high degree of measurement accuracy, the following scanning (iCAT next generation, Imaging Sciences International (ISI), Hatfield, PA, USA) parameters were chosen: 120KvP, 5 mA, voxel size 0.25 mm, 14.7 s acquisition time, high-definition scan mode of 360° (total rotation), field of view (FOV diameter 16 cm), height 6 cm with a resolution of 0.157 × 0.157 mm. Each patient’s three-dimensional volumetric pictures that were captured and rebuilt were exported as DICOM files (Digital Imaging and Communications in Medicine) and examined with an image analysis program (Ondemand3D App v1.0.10.7510, CyberMed, Korea) [27].

**Fig. 3:**
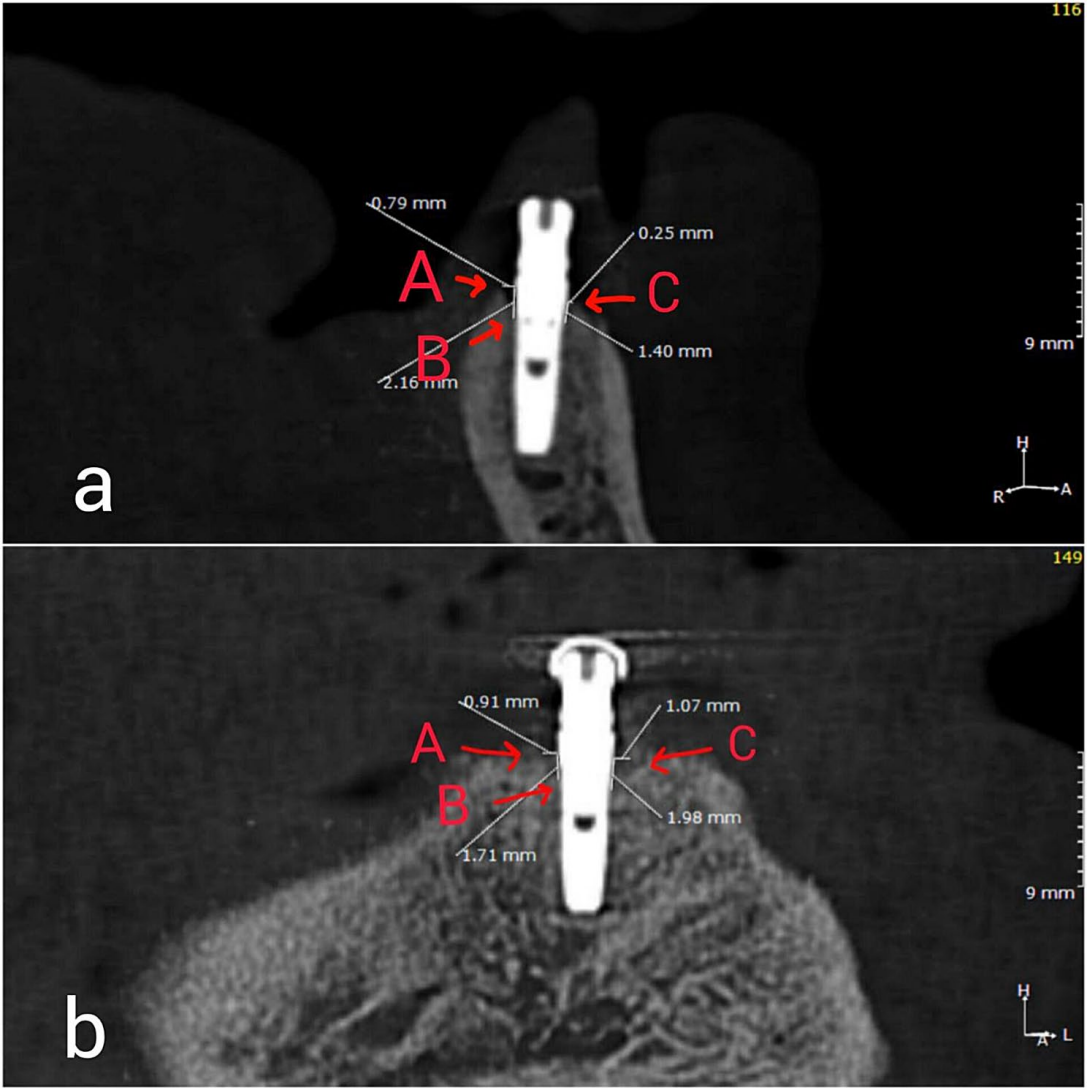
Measurements of the peri-implant bone loss. **a**: Buccolingual image. **b**: Mesiodistal image. A: the distance between the attachment-implant junction. B: the initial bone-implant contact. C: the marginal bone crest

The position of the dental implant in the patient’s mouth was determined using a three-dimensional system, which located the center of the implant’s coronal portion. Then, two vertical transversal images were produced using digital guidelines to create horizontal planes at right angles to the implant’s long axis. These images included a buccolingual image, which was formed by the implant’s buccolingual bisectional axis, and a mesiodistal image, which was formed by bisecting the alveolar crest and implant mesiodistally. Each image recognized the four faces surrounding the implant: buccal, lingual, distal, and mesial [28, 29]. The vertical and horizontal bone levels, also known as marginal bone levels, were then established on all four faces. The vertical bone level was calculated by measuring the distance between the component-implant junction (A) and the initial bone-implant contact (B) in millimeters. The horizontal bone level was calculated by measuring the perpendicular distance (in mm) between the implant and the marginal bone crest (point C). (Fig. 3)

Measurements of vertical bone loss (VBL) and horizontal bone loss (HBL) were taken at three different time intervals: implant insertion (T0), one year later (T1), and five years later (T5). Positive values indicate bone loss, while negative values indicate bone gain or apposition [26, 27]. The mean bone level was determined by averaging the measurements of both vertical and horizontal levels across all implant faces. The measurements were conducted twice with standardized image contrast and brightness to ensure accuracy. A single-blinded calibrated evaluator performed the measurements in random order and in duplicate to calculate the reliability index of the measurements, and the statistical analysis was based on the mean of two implants.

### Evaluation of the mandibular posterior ridge resorption

The linear dimensional measurements for posterior ridge resorption (PRR) were carried out on the same CBCT scans that were used to determine the circumferential bone level, following the method discussed by Schuster et al. [27]. The OnDemand software was used for post-processing, with a cut thickness of 0.5 mm. To standardize the measurements for different time intervals, a standardized slice was selected for the same patient from the different CBCT images using the fusion module of the OnDemand software (Ondemand3D App v1.0.10.7510, CyberMed). The posterior region of the mandible was identified by the location of the mental foramen opening, and the digital reference lines were positioned perpendicular to the posterior wall of the foramen and centered on the ridge in the buccal-lingual direction. This approach generated a sagittal cut that identified the entire posterior region of the mandible at its maximum height by using the reference lines in the central region of the ridge in the mandibular canal.

**Fig. 4:**
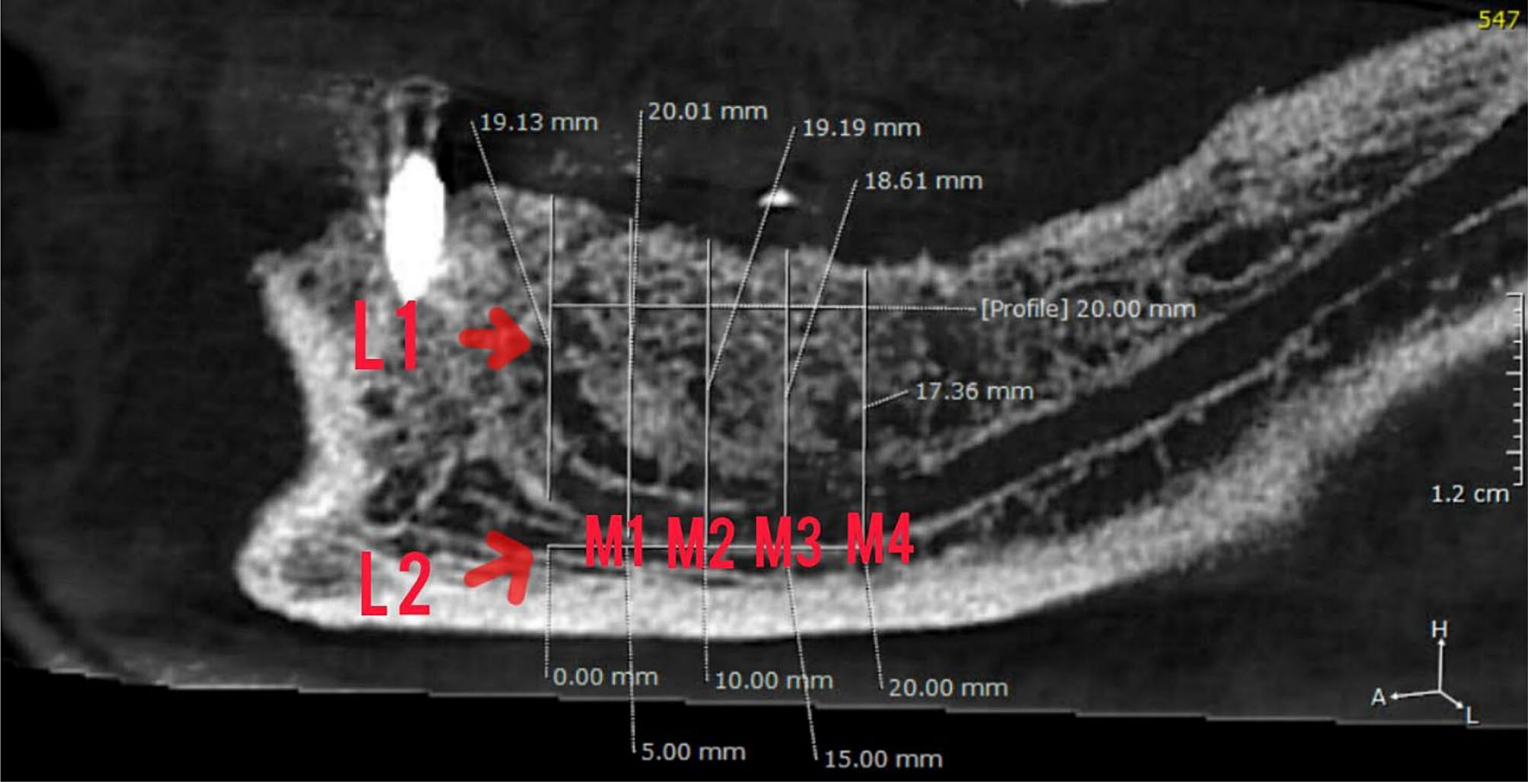
Measurements of posterior alveolar ridge resorption. L1: a perpendicular line from the longitudinal posterior wall of the mental foramen to the base of the mandibular canal. L2: reference lines from the horizontal liner of the right angle. M1, M2, M3, and M4: parallel lines from the crest of the ridge to the horizontal line at the base (L2) at distances of 5, 10, 15, and 20 mm from the mental foramen, respectively

Following the necessary adjustments, a perpendicular line was drawn from the longitudinal posterior wall of the mental foramen to the base of the mandibular canal (L1). To obtain linear bone measurements at specific intervals of 5, 10, 15, and 20 mm (L2), reference lines were drawn from the horizontal line of the right angle. Additionally, a parallel line was drawn from the crest of the ridge to the horizontal line at the base of the mandibular canal, which was formed by the vertical line from the right angle. The posterior mandibular region’s total ridge height was then measured at four points (M1, M2, M3, and M4) along this line, located at distances of 5, 10, 15, and 20 mm from the mental foramen, respectively (Fig. 4).

### Statistical analysis

Data were analyzed using the Statistical Package of Social Science (SPSS) program for Windows (Standard version 26). A non-normally distributed data was accessed using Shapiro-Wilk test. Mann-Whitney test was used to compare two different groups. Paired groups were compared using Wilcoxon signed rank test. The threshold of significance is fixed at a 5% level (p-value). The results were considered significant when the *p* < 0.05. The smaller the p-value obtained, the more significant the results.

## Results

A comparison of the differences in vertical bone loss (VBL) between the two groups is presented in Table 1. There was no significant difference in VBL between groups at the time (T1-T0) (Mann Whitney test, *P* = 0.088), while significant differences between groups appeared at (T5-T1) (Mann Whitney test, *P* = 0.01) and at (T5-T0) (Mann Whitney test, *P* = 0.005). VBL values decreased in group L compared to group C, as the canine group recorded higher values of VBL at T1 and T5 compared to the lateral incisors group.

Descriptive statistics of differences in VBL within each group at the different follow-up periods are presented in Table 2. There was no significant difference in VBL in group L or group C over follow-up periods.

**Table 1:** Vertical bone loss among the studied groups

Vertical bone loss	Group (L) (n = 25)	Group (C) (n = 25)	Test of significance	P value
T1-T0				
Mean ± SD	-0.148 ± 0.33	0.037 ± 0.21	Z = 1.70	0.088
Median	-0.034	0.097		
Min-Max	-0.94- 0.13	-0.32- 0.28		
T5-T1				
Mean ± SD	-0.067 ± 0.18	0.109 ± 0.76157	Z = 2.57	0.01*
Median	-0.005	0.336		
Min-Max	-0.53- 0.09	-1.98- 0.70		
T5-T0				
Mean ± SD	-0.216 ± 0.41	0.146 ± 0.70	Z = 2.80	0.005*
Median	-0.087	0.237		
Min-Max	-1.05- 0.21	-1.91- 0.48		

Z: Mann Whitney test, *significant p ***<*** 0.05

**Table 2:** Comparison of Vertical bone loss at different follow-up periods

Vertical bone loss	T1-T0	T5-T1	T5-T0	Wilcoxon signed-rank test		
				P1	P2	P3
Group (L) Median (Min-Max)	-0.034 (-0.94- 0.13)	-0.005 (-0.53- 0.09)	-0.087 (-1.05- 0.21)	Z = 0.71 P = 0.48	Z = 1.26 P = 0.21	Z = 1.28 P = 0.20
Group (C) Median (Min-Max)	0.097 -0.32- 0.28	0.336 -1.98- 0.70	0.237 -1.91- 0.48	Z = 1.07 P = 0.28	Z = 1.37 P = 0.17	Z = 0.66 P = 0.51

Z: Wilcoxon signed-rank test, *significant p ***<*** 0.05, P1: (T1-T0) vs. (T5-T1), P2: (T1-T0) vs. ( T5-T0), P3: (T5-T1) vs. (T5-T0)

A comparison of the differences in horizontal bone loss (HBL) between groups is presented in Table 3. Significant differences in HBL between groups were found at (T1-T0) (Mann Whitney test, *P* = 0.041) and (T5-T1) (Mann Whitney test, *P* = 0.041), while no significant differences in HBL between groups appeared at (T5-T0) (Mann Whitney test, *P* = 0.880). Group L showed lower HBL values over time (T1-T0) and (T5-T1) as compared to group C, while the HBL values for both groups were comparable.

**Table 3:** Horizontal bone loss among the studied groups

Horizontal bone loss	Group (L) (n = 25)	Group (C) (n = 25)	Test of significance	P value
T1-T0				
Mean ± SD	-0.060 ± 0.27	0.359 ± 0.43	Z = 2.04	0.041*
Median	0.015	0.185		
Min-Max	-0.80- 0.18	-0.14- 0.97		
T5-T1				
Mean ± SD	0.013 ± 0.27	-0.851 ± 0.81	Z = 2.04	0.041*
Median	-0.04	-0.97		
Min-Max	-0.26- 0.61	-1.92- 0.40		
T5-T0				
Mean ± SD	-0.047 ± 0.15	-0.492 ± 0.88	Z = 0.151	0.880
Median	-0.035	-0.02		
Min-Max	-0.21- 0.23	-1.88- 0.37		

Z: Mann Whitney test, *significant p ***<*** 0.05

Descriptive statistics of differences in HBL within each group at different follow-up periods are presented in Table 4. There was no significant difference in HBL in group L over the follow-up periods, while in group C there was a significant difference in HBL over time between each evaluation period.

**Table 4:** Comparison of horizontal bone loss at different follow-up periods

Horizontal bone loss	T1-T0	T5-T1	T5-T0	Wilcoxon signed rank test		
				P1	P2	P3
Group (L) Median (Min-Max)	0.015 (-0.80- 0.18)	-0.04 (-0.26- 0.61)	-0.035 (-0.21- 0.23)	Z = 0.56 *P* = 0.57	Z = 0.41 *P* = 0.68	Z = 0.18 *P* = 0.85
Group (C) Median (Min-Max)	0.185 (-0.14- 0.97)	-0.97 (-1.92- 0.40)	-0.02 (-1.88- 0.37)	Z = 2.39 P = 0.017*	Z = 2.29 P = 0.022*	Z = 2.29 P = 0.022*

Z: Wilcoxon signed rank test, *significant *p* < 0.05, P1: (T1-T0) vs. (T5-T1), P2: (T1-T0) vs. ( T5-T0), P3: (T5-T1) vs. (T5-T0)

A comparison of posterior ridge resorption (PRR) between the studied groups is presented in Table 5. There were significant differences over the evaluation period between groups at M1 (Mann Whitney test, *P* = 0.021), M3 (Mann Whitney test, *P* = 0.008), and M4 (Mann Whitney test, (*P* = 0.015). At the same time, there was no significant difference at M2 (Mann-Whitney test, *P* = 0.094). Group C recorded higher PRR values over the evaluation period as compared to group L.

**Table 5:** Posterior ridge resorption among the studied groups

Posterior ridge resorption	Group (L) (n = 25)	Group (C) (n = 25)	Test of significance	P value
M1 T5-T0				
Mean ± SD	-0.374 ± 0.79	-0.711 ± 0.19	Z = 2.31	0.021*
Median	-0.027	-0.635		
Min-Max	-2.32-0.10	-1.25–0.60		
M2 T5-T0				
Mean ± SD	-0.607 ± 1.03	0.364 ± 0.26	Z = 1.68	0.094
Median	-0.09	-0.20		
Min-Max	-2.84-0.03	-0.85–0.20		
M3 T5-T0				
Mean ± SD	-0.502 ± 1.15	-0.766 ± 0.39	Z = 2.65	0.008*
Median	-0.10	-0.552		
Min-Max	-3.76–0.02	-1.35–0.42		
M4 T5-T0				
Mean ± SD	-0.433 ± 1.27	-0.692 ± 0.42	Z = 2.42	0.015*
Median	-0.07	-0.505		
Min-Max	-3.98-0.30	-1.50–0.44		

Z: Mann Whitney test, *significant p ***<*** 0.05

Descriptive statistics of differences in PRR between different points within each group are presented in Table 6. For group L, there was no significant difference in PRR between the evaluated points over the evaluation period. In contrast, group C showed significant differences between (M1 vs. M2) (Mann Whitney test, P1 = 0.015), (M1 vs. M4) (Mann Whitney test, P3 = 0.023), (M2 vs. M3) (Mann Whitney test, P4 = 0.004), and (M2 vs. M4) (Mann Whitney test, P5 = 0.009).

**Table 6:** Comparison of posterior ridge resorption at different follow-up periods

Posterior ridge resorption	M1 T5-T0	M2 T5-T0	M3 T5-T0	M4 T5-T0
Group (L) Median (Min-Max)	-0.027 (-2.32-0.10)	-0.09 (-2.84-0.03)	-0.10 (-3.76–0.02)	-0.07 -3.98-0.30
P value	P1 = 0.58, p2 = 0.29, p3 = 0.73, p4 = 0.62, p5 = 0.41, p6 = 0.49			
Group (C) Median (Min-Max)	-0.635 (-1.25–0.60)	-0.20 (-0.85–0.20)	-0.552 (-1.35–0.42)	-0.505 -1.50–0.44
P value	P1 = 0.015*, p2 = 0.65, p3 = 0.023*, p4 = 0.004*, p5 = 0.009*, p6 = 0.76			

Mann Whitney test was used, *significant p < 0.05

p1: M1 T5-T0 vs. M2 T5-T0,

p2: M1 T5-T0 vs. M3 T5-T0,

p3: M1 T5-T0 vs. M4 T5-T0,

p4:. M2 T5-T0 vs. M3 T5-T0,

p5: M2 T5-T0 vs. M4 T5-T0,

p6: M3 T5-T0 vs. M4 T5-T0

## Discussion

The current study assessed the changes in peri-implant bone levels and resorptive changes in the posterior mandibular ridge for patients with mandibular implant overdentures in two groups, namely Group L and Group C. This evaluation was conducted using CBCT scans taken at 1- and 5-years post-insertion. The findings indicated significant differences in vertical and horizontal bone loss between the groups. Moreover, there were significant differences in posterior ridge resorption over the evaluation period at specific points along the ridge. Consequently, the null hypothesis, which stated that there are no differences between the two implant positions concerning peri-implant bone loss or resorptive changes in the posterior mandibular ridge, was rejected.

It was revealed that an overload of implants is the primary factor behind bone resorption, which directly impacts bone due to elevated functional load [14, 30]. In cases where patients are completely edentulous, the anterior mandible typically retains more residual alveolar bone, making it an ideal site for implants [31, 32]. When there is an adequate amount of alveolar bone present, it is recommended to insert two implants in the canine areas. However, if the level of alveolar bone in the lateral incisor and canine positions is comparable, the lateral incisor areas may be a more appropriate choice [33]. Resilient attachment; like that used in this study; was found to occupy a greater denture/mucosa contact area compared to rigid attachment [34]. Moreover, it was observed that using resilient attachments for two-implant overdentures may result in less peri-implant bone stress, but the highest concentration and extent of alveolar ridge bone stress were found in the posterior mandible regions. These findings suggested observing the posterior ridge resorptive changes with implant overdentures, especially for patients who may benefit from anterior-placed implants.

In the present study, peri-implant bone changes and posterior ridge resorptive changes were evaluated using CBCT.CBCT provides volumetric information, which is medically important for assessing the patient’s anatomy and the possible bony changes [35]. In previous studies [22, 36], it was used to monitor the ridge base relation and in other studies [27, 37], it was used to evaluate alveolar bone height and its loss. Compared to conventional CT systems, CBCT provides an accurate 3D image with minimal radiation exposure [38].

The results of peri-implant bone loss evaluation revealed that there was no significant difference in VBL between the lateral incisor and canine groups at (T1-T0). However, after a longer evaluation period at (T5-T1), a significant difference between the two groups appeared. This may be because the longer evaluation period allowed for crestal bone changes to be recognized. The results showed that the lateral incisor group had a decrease in VBL compared to the canine group. At T1 and T5, the canine group had higher VBL values than the lateral incisor group. Furthermore, there was a significant difference between the two groups in the HBL at (T1-T0) and (T5-T1). The canine group only showed a significant difference in the HBL over time between each evaluation period.

Considering these results from a biomechanical point of view, studies found that a two-implant overdenture tends to rotate easily around the fulcrum line which is formed between the two implants, forming the anterior and posterior lever arm, when an anterior loading is applied [39, 40]. While the denture-bearing area of the molar region is larger than that of the anterior region and resisted occlusal force more strongly. Therefore, the two-implant overdenture hardly rotates posteriorly under occlusal forces [40]. So, implants placed more anteriorly in the position of the lateral incisors could allow for a shorter anterior lever arm with more support and retention anteriorly by the implants and the attachments. This would allow for a decreased anterior rotational movement and subsequently less lateral stresses as compared to the canine group and could explain the decrease in VBL values in the lateral incisors group in comparison to the canine group.

Evaluating the posterior ridge resorptive changes was compared at the time of insertion (T0) and 5 years after insertion (T5) to observe the bony changes that may occur after insertion of the implant overdenture of each group. The comparison of posterior ridge resorption between group L and group C revealed a significant difference at multiple points along the posterior edentulous area, specifically at M1, M2, and M3. Furthermore, group C showed higher PRR values over the evaluation period in comparison to the lateral incisors group. This can be attributed to the difference in the surface area of contact between the denture and mucosa in the two groups. In group L, more anterior implant placement allowed for a greater surface area of the edentulous ridge which shared the support and distributed stresses over a larger surface area. Similar results were found by Liu J. et al., [39] in their in-vitro study, where anterior implant placement that allowed more contact area between the denture and mucosa reduced pressure on the mucosa of the edentulous ridge. Furthermore, they observed reduced maximum strain values in the peri-implant bone, which is consistent with the less peri-implant bone loss seen in the lateral incisors group in this study. The implants in the lateral incisors group appear to take the role of retention rather than support.

In agreement with the results of the current study, Hong et al. [11] in their study suggested that in implant-retained overdenture procedures, lateral incisors are the optimal location for implant placement. This is due to the decreased level of peri-implant stress found in this area, which directly contributes to implant longevity and support. When subjected to both vertical and horizontal loads, implants in the anterior region result in a more even stress distribution and anteroposterior stability. In-vitro experiments corroborate these findings, indicating that models with implants placed in the lateral incisor areas exhibit the lowest levels of peri-implant stress and the most efficient stress distribution [11, 41].

In contrast to the findings of this study, Alvarez-Arenal A. et al. [9] did not recommend placing implants in the position of lateral incisors for implant overdentures. According to their research, the lateral incisor was found to have the most unfavorable biomechanical environment, and the crossed-implant overdenture models had maximum peri-implant bone stress levels of 38.65 MPa and 43.89 MPa, respectively. However, it is worth noting that these stress levels are still within the bone’s physiological tolerance threshold [42, 43]. The difference in results between this study and Alvarez-Arenal A.‘s study could be attributed to their in-vitro study not considering the complex force patterns of varying magnitude and direction that occur during chewing in different areas of the mouth, which could produce different stress patterns that are difficult to mathematically reproduce and simulate.

Of particular significance in this study is the observation that group C, wherein implants were inserted in the canine position, exhibited notable variations in bone resorption across different locations along the residual ridge. The investigation revealed a marked escalation in bone loss values as the points moved towards the posterior end. Conversely, group L, where implants were placed in the lateral incisors position, showed no significant differences in bone loss across various locations along the ridge. These findings imply that anterior implants offer superior anterior support, enabling greater posterior vertical force application with better bone response. Another clinical study recommended placement for implants in the lateral incisor area as close to the anterior as possible to minimize the posterior rotation and allow for a greater denture/mucosa contact area [10]. Nonetheless, it’s crucial to verify that the denture base is properly fitted on the edentulous ridge and is appropriately extended. Moreover, it has also been suggested that overdentures retained with anterior implants should not have definite occlusal contacts in the anterior area. This is to minimize vertical movement that may occur mainly in this type of prosthesis when there is anterior loading during incising food [40].

The study’s findings validate prior research indicating that implant placement in the lateral incisor position offers significant benefits [11, 44]. This is due to the favorable anatomical characteristics within this region, including a higher trabecular bone ratio and thicker cortical bone [45, 46]. Furthermore, this strategic placement reduces tipping forces on the overdenture through a decreased effective anterior level arm, leading to enhanced stability. Also, Vogel stated that placing implants closer to the lateral incisor position also allows for additional implant placements if needed, making the patient an ideal candidate for an All-on-4 implant prosthesis [47]. Additionally, implant placement in this position leads to less of a “seesaw effect” and higher patient satisfaction. In cases of advanced alveolar bone resorption, implant placement opportunities are limited, requiring extensive surgical reconstruction procedures. Placing implants in the anterior area, where such procedures are less frequently needed, can help avoid complications and reduce stress on both the patient and the operator [48].

A potential constraint of the research is the restricted number of patients caused by dropouts and stringent inclusion criteria. The study mandated that all patients receive treatment from a single surgeon and utilize identical implants and prosthetic components to ensure the standardization of several variables.

## Conclusion

According to the findings of this clinical study, the placement of implants in the lateral incisor position for two implant-retained overdentures is a viable choice. In comparison to the canine position, the lateral incisor position demonstrated superior peri-implant responses, which could potentially enhance the longevity of the implants. Furthermore, the placement of implants in the lateral incisor position can promote a more even distribution of stress and help mitigate posterior ridge resorption. Conversely, implants in the canine position may cause a seesaw effect and result in greater posterior ridge resorption.

## Data Availability

The datasets used in the current study are available from the corresponding author upon request.
